# A novel optimized silver nitrate staining method for visualizing the osteocyte lacuno-canalicular system

**DOI:** 10.3389/fendo.2025.1561576

**Published:** 2025-05-15

**Authors:** Jinlian Wu, Chunchun Xue, Qiang Li, Hongjin Wu, Jie Zhang, Chenglong Wang, Weiwei Dai, Libo Wang

**Affiliations:** ^1^ Central Laboratory of Science and Technology, Longhua Hospital Shanghai University of Traditional Chinese Medicine, Shanghai, China; ^2^ Department of Rheumatology and Immunology, Longhua Hospital Shanghai University of Traditional Chinese Medicine, Shanghai, China; ^3^ Shanghai Municipal Hospital of Traditional Chinese Medicine, Shanghai University of Traditional Chinese Medicine, Shanghai, China; ^4^ Longhua Hospital Shanghai University of Traditional Chinese Medicine, Shanghai, China

**Keywords:** silver nitrate, gelatin, formic acid, osteocyte, lacuno-canalicular system (LCS)

## Abstract

A 50% (w/v, equivalent to 2.943 mol/L) silver nitrate solution is commonly used to stain and characterize the osteocyte lacuno-canalicular system (LCS) in bone biology research. However, variability in reagent concentrations and types, along with inconsistent staining procedures, have limited the broader application of this method in osteocyte research. In this study, we present a novel optimized silver nitrate staining technique aimed at addressing these limitations. This new method utilizes a 1 mol/L (equivalent to 16.987%, w/v) silver nitrate solution in combination with a type-B gelatin-formic acid solution at various concentrations (0.05%-0.5% gelatin and 0.05%-5% formic acid, or 1%-2% gelatin and 0.1%-2% formic acid) in volume ratios of 4:1, 2:1, or 1:1, or a 0.5 mol/L silver nitrate solution at a 4:1 ratio. The staining process is carried out for 1 hour under ultraviolet light or 90 minutes under regular room light (or dark), followed by washing with Milli-Q water to terminate the reaction. We applied this new method to stain the osteocyte LCS in bone samples from different species and pathological bone models. The technique consistently produced clear, distinct staining patterns across all samples. Moreover, our novel method revealed a greater number of LCS compared to the traditional 50% silver nitrate solution. This suggests that the commonly used 50% silver nitrate method may disrupt or inadequately reveal the LCS in bones, potentially leading to an underestimation of LCS density and number. In conclusion, our novel silver nitrate staining method provides a simpler and more cost-effective alternative to the traditional technique. By offering a more accurate and comprehensive analysis of the LCS across species, this approach has the potential to advance research on osteocyte morphogenesis, as well as the functional and evolutionary adaptations of the osteocyte LCS across different taxa.

## 1 Introduction

Osteocytes are terminally differentiated osteoblasts embedded in the bone matrix within small cavities called lacunae. A hallmark of osteocytes is their dendritic processes—long, branching protrusions that extend from the cell body. These dendrites pass through tiny canals called canaliculi, which connect the lacunae throughout the bone matrix (1–3). This network of lacunae and canaliculi, known as the lacuno-canalicular system (LCS), along with the dendritic network, exhibits a remarkable and intricate architecture similar to the dendritic connectivity observed in neuronal cells (4, 5). The adult human skeleton contains approximately 42 billion osteocytes and 3.7 trillion dendrites, with each osteocyte having 18 to 106 dendrites that extend into 53 to 126 canaliculi connected to the lacunae (4).

The osteocytes and their LCS are critical for several physiological functions, including nutrient and waste exchange, as well as cell signaling between osteocytes and other cells, such as osteoblasts, osteoclasts, and vascular endothelial cells (6–9). Given the central role of the LCS in bone biology, extensive research has been dedicated to understanding its structural and functional changes in various contexts, including bone development, bone diseases (e.g., osteoporosis, osteoarthritis), pregnancy, and aging (10–14).

Among the various histological staining techniques, the silver nitrate method, particularly the Ploton method developed in 1986, is widely used to investigate the osteocyte LCS (15–17). This technique is effective in revealing the structure of the LCS, allowing for detailed analysis of osteocyte connectivity. However, despite its utility, the method has several limitations. These include inconsistencies in the concentrations of silver nitrate and gelatin-formic acid solutions, a lack of standardization in staining protocols, variability in the type of gelatin used, and inconsistencies in staining and termination conditions. These factors have impeded the wider adoption of Ploton method in osteocyte research.

In this study, we developed a simplified, more environmentally friendly silver nitrate staining method that improves upon the classical method that uses a 50% silver nitrate solution. Our approach used a 1 mol/L silver nitrate solution combined with type-B gelatin-formic acid solutions at various concentrations (0.05%-0.5% gelatin and 0.05%-5% formic acid, or 1%-2% gelatin and 0.1%-2% formic acid) in volume ratios of 4:1, 2:1, or 1:1, or a 0.5 mol/L silver nitrate solution at a 4:1 ratio. The staining was performed for 1 hour under UV light or 90 minutes under regular room light (or dark). After staining, the slides were washed with Milli-Q water (no need for 5% Sodium thiosulfate), followed by standard dehydration, clearing, and mounting procedures. The novel optimized method yielded consistent and distinct staining across different species and disease models, demonstrating superior efficiency in staining osteocyte LCS at higher densities and greater numbers compared to the Ploton method. We believe that our new method will contribute to advancing the study of bone biology and have broader applications in the diagnosis and treatment of bone-related diseases.

## 2 Materials and methods

### 2.1 Animal bone sample collection

The Male C57BL/6J mice (4–8 weeks old) and Sprague-Dawley rat (8 weeks old) were purchased from Hangzhou Ziyuan Laboratory Animal Technology Co., Ltd (Hangzhou, China). Prrx1Cre mice and Zeb1^fx/fx^ mice were purchased from GemPharmatech Co., Ltd (Jiangsu, China). All mice and rat were housed under standard conditions at the animal facility of Shanghai Municipal Hospital of Traditional Chinese Medicine. Rabbit (*Oryctolagus cuniculus*), common carp (*Cyprinus carpio*), silver carp (*Hypophthalmichthys molitrix*), snakehead (*Channa maculata*), little yellow croaker (*Larimichthys polyactis*), bullfrogs (*Lithobates catesbeiana*), chickens (*Gallus gallus domesticus*), and wall lizards (*Gekko* sp.) were purchased from a local food market and an online traditional Chinese medicine herb store. A bone growth retardation model was created by administering dexamethasone (2 mg/kg/day) intraperitoneally to 4-week-old male mice for four weeks. A transgenic mouse model (Prrx1Cre;Zeb1*^fx/fx^* ^)^, which exhibits a pathological phenotype characterized by cartilage remnants in the cortical bone region of adult mice and specifically overexpresses Zeb1 in skeletal mesenchymal stem cells, was generated by crossing Prrx1Cre mice with Zeb1^fx/fx^ mice. Femurs from mice and rats were harvested by carbon dioxide (CO_2_) inhalation, which was performed at a flow rate of 50% of the chamber volume displacement per minute. Femurs from bullfrogs, chickens, rabbits and wall lizards, as well as caudal vertebrae from common carp, silver carp, snakehead, and little yellow croaker, were isolated upon purchase. Animal experiments were performed in strict compliance with the guidelines and were approved by the Ethics Committee of Experimental Animal Medicine of Shanghai Municipal Hospital of Traditional Chinese Medicine, Shanghai University of Traditional Chinese Medicine (Approval No. 2020007).

### 2.2 Reagents and apparatus

Reagents:10% EDTA Decalcification solution (E671001-0500, Sangon Biotech), 10% Neutral formalin fixative (311010014, Wexis), 1 mol/L Silver nitrate solution (P1929026, Bolinda Technology), Silver nitrate power (C510027-0010, Sangon Biotech), Type-A Galetin (A609764-0100, Bloom 238~282, Sangon Biotech), Type-B Galetin (G8061, bloom 225, Solarbio), 88% Formic acid (10010118, SinoPharm), Dexamethasone (HY-14648, MedChemExpress), Sodium thiosulfate (A600484-0500, Sangon Biotech), Neutral balsam mounting medium (G8590, Solarbio), Ethanol (Sinopharm, catalog number: 10009218), Xylene (Sinopharm, catalog number: 10023418), Paraffin (Leica, catalog number: 39601006). Apparatus: Biological Safety Cabinet(1379/1389 with 254nm UV light G3675L 31~40W, Thermo), Automatic Benchtop Tissue Processor (TP1020, Leica), Tissue Embedding Station (EG1160, Leica), Rotary microtome (RM2135, Leica), Histology Water Bath (Histobath HI1210, Leica), Ultrapure Water Purification System (Milli-Q Elix^®^ Essential, Millipore), Nikon bright-field microscope (ECLIPSE 200, Nikon).

### 2.3 Bone sample processing

All animal bone samples were fixed in 10% neutral formalin for 24 hours, followed by washing in 18.2 MΩ·cm Milli-Q water six times, each lasting 10 minutes. The bones were then decalcified in 10% EDTA (pH 7.4) at room temperature for 28 days, with the solution being replaced every three days. After decalcification, the bones were dehydrated through a graded ethanol series (70%, 80%, and 90% for 1 hour each, followed by 100% ethanol three times for 1 hour each), cleared in a graded xylene series (50:50 ethanol-xylene for 1 hour, then 100% xylene twice for 1 hour each), and infiltrated with molten paraffin at 60°C three times. The bones were then embedded in paraffin. When preparing sections of the femur or tibia, the bones were sliced along the longitudinal axis. Bone sections with a thickness of 4 μm were collected when two complete, parallel coronal profiles were exposed on the longitudinal section, typically at a depth of one-third to one-half of the total cutting depth. The sections were dried in a thermoelectric oven at 37°C for 1 hour to adhere well to the slides, dewaxed at 60°C for 6 hours to remove wax and residual moisture completely, and then stored at room temperature or 4°C for long-term preservation.

### 2.4 Silver nitrate staining

Solution preparation: (1) 1 mol/L Silver nitrate solution: Prepare the silver nitrate solution using 18.2 MΩ·cm Milli-Q water. Store the solution at room temperature, protected from light. You can also buy pre-made 1 mol/L silver nitrate solution online, which is widely available and commonly used in chemical titration experiments. (2) Gelatin-Formic acid solution: Prepare a 1% (v/v) formic acid solution using 18.2 MΩ·cm Milli-Q water. Weigh 2% (w/v) type-B gelatin and add it to the formic acid solution. Heat the mixture at 37°C for 2 hours, or until the gelatin is fully dissolved. Store at room temperature. Using our novel method, a gelatin-formic acid solution can be prepared with either 0.05%–1% (w/v) type-B gelatin and 0.05%–5% (v/v) formic acid or 1%–2% (w/v) type-B gelatin and 0.1%–2% (v/v) formic acid, achieving staining results as clear and effective as a 2% (w/v) type-B gelatin in 1% formic acid solution. (3) Staining solution: Combine two volumes of the 1 M silver nitrate solution with one volume of the 2% type-B gelatin-1% formic acid solution. Prepare the staining solution immediately before use. Prepare the solution immediately before use, as pre-preparing may lead to poor or incomplete staining. Using our novel method, the volume ratio of silver nitrate to the gelatin-formic acid solution can be 2:1 or 1:1.

**Figure 1:**
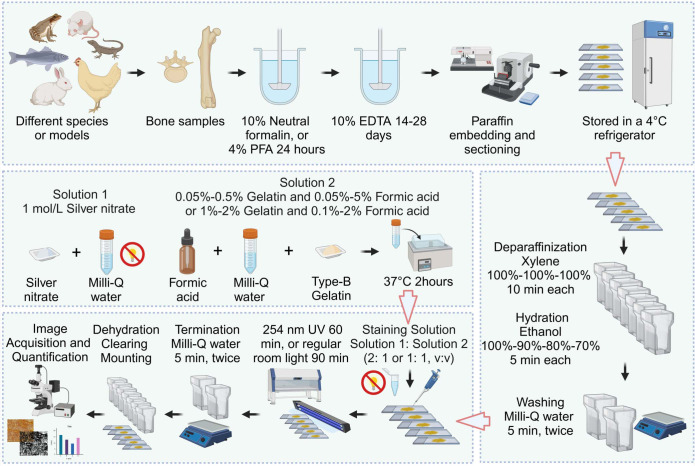
Flowchart of the Novel Optimized Silver Nitrate Staining Method. This schematic outlines the key steps for staining the osteocyte LCS. Created in BioRender. Libo, W. (2025) https://BioRender.com/m59m214.

Notes: (1) Storage Duration of Gelatin-Formic Acid: Prolonged storage of gelatin-formic acid solutions can result in suboptimal staining performance. We recommend using freshly prepared solutions within 3 months. If a solution has been stored for over 3 months, extend the staining time to achieve the best results. (2) Reactivity of Silver Nitrate Solution: Silver nitrate solutions react with tap water, PBS, TBS, or any salt-containing solutions, forming a white precipitate. To avoid interference with staining quality, rinse slides with Milli-Q water after rehydration and staining. (3) Freshly Prepared Staining Solution: The staining solution is initially clear when prepared but turns yellow within 5–10 minutes. Yellowed solutions are much less effective for LCS staining, so it is essential to apply the solution to slides as soon as possible. (4) Type of Gelatin: Use Type-B gelatin instead of Type-A, as Type-A gelatin can lead to the formation of numerous black silver particle precipitates on stained slides.

Staining procedure: Following deparaffinization with 100% xylene (10 minutes at room temperature, repeated twice for a total of three changes) and rehydration through a graded ethanol series (100%, 90%, 80%, and 70%, 5 minutes each), paraffin sections are washed twice in 18.2 MΩ·cm Milli-Q water for 5 minutes each. Next, two volumes of a 1 mol/L silver nitrate solution are mixed with one volume of a 2% (w/v) type-B gelatin-1% (v/v) formic acid solution and applied to the slides. The slides are then stained for 60 minutes under 254 nm ultraviolet light or 90 minutes under regular room light at room temperature. After staining, the reaction is stopped by washing the slides twice in 18.2 MΩ·cm Milli-Q water for 5 minutes each, followed by dehydration (70%, 80%, 90%, and 100% ethanol, 5 minutes each), clearing (100% xylene, three times for 10 minutes each), and mounting with neutral balsam. The general procedure for the optimized silver nitrate staining method for osteocyte LCS is shown in **Figure 1**.

### 2.5 LCS quantification

The osteocyte LCS area was quantified and normalized to the total bone area as previously described (18, 19). Images at 400× magnification were acquired using a Nikon ECLIPSE 200 bright-field microscope (Nikon, Japan). The quantification process using ImageJ software (NIH, USA) for LCS staining is as follows: Open the image in ImageJ (*Image > Open*). For RGB images, split the image into individual channels (*Image > Color > Split Channels*) and select the green channel for analysis. Enhance contrast by going to *Process > Enhance Contrast*, setting “*Saturated pixels*” to 0.3% (default) and optionally selecting “*Equalize histogram*.” Apply a threshold to distinguish positive LCS staining from the background (*Image > Adjust > Threshold*) using ImageJ’s default values without manual adjustments to ensure consistency. Define the region of interest (ROI) using the *Polygon* selection tool, then set measurement parameters (*Analyze > Set Measurements*) by selecting *Area*, *Integrated density*, *Area fraction*, *Mean gray value*, and *Limit to threshold*. Measure the positive staining area (*% Area*) (*Analyze > Measure*) and repeat the process for all images.

### 2.6 Data reproducibility and statistics analysis

**Figure 2:**
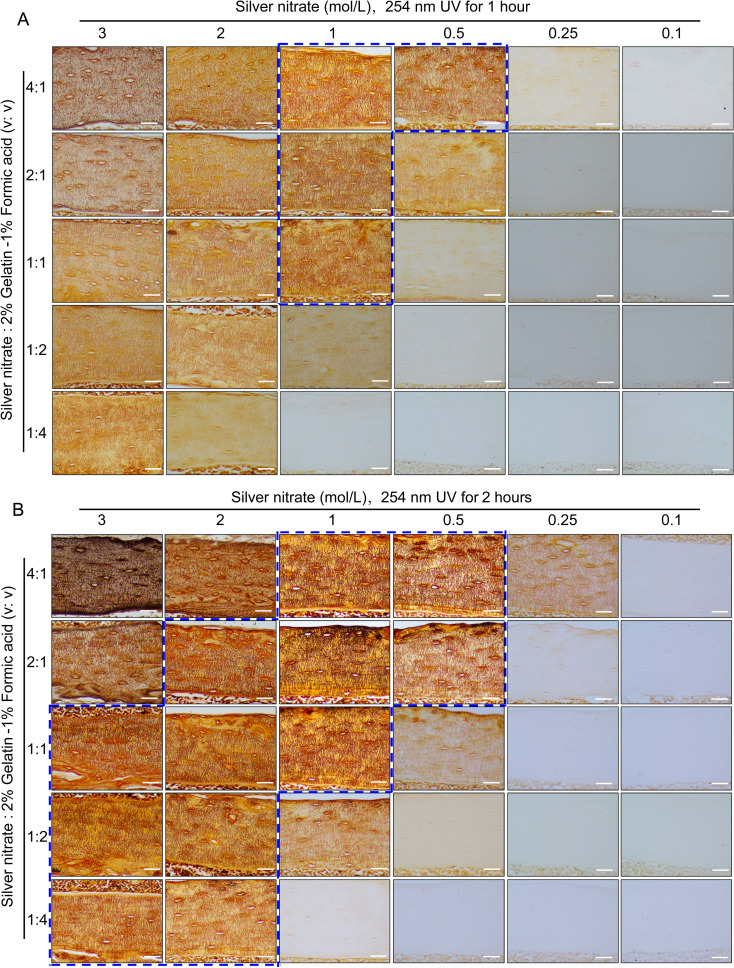
Staining effects of different concentrations of silver nitrate combined with different volume ratios with 2% gelatin-1% formic acid solution when staining under UV 1hour or 2 hours on LCS. **(A)** Effects of exposure to 254 nm UV light for 1 hour to stain LCS. Scale bars = 50 μm. **(B)** Effects of exposure to 254 nm UV light for 2 hours to stain LCS. Scale bars = 50 μm. The dashed blue box indicates the optimal silver nitrate concentration and the volume ratio of silver nitrate solution to type-B gelatin-formic acid solution for clear and effective staining of osteocyte LCS. All scale bars = 50 μm.

**Figure 3:**
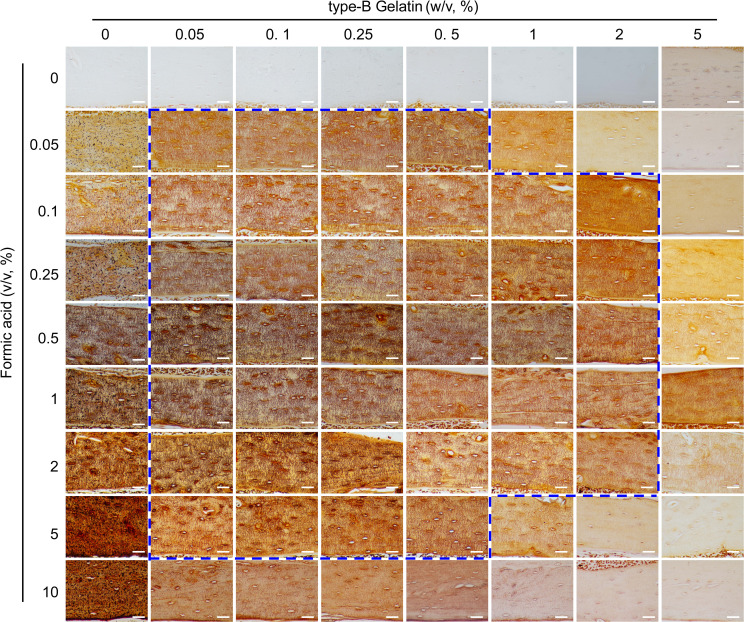
Comparison of the staining effects on LCS using gelatin (0%-5%, w/v)-formic acid (0%-10%, v/v) solutions at different concentrations, in a 2:1 volume ratio with 1 mol/L silver nitrate. The dashed blue box indicates the effective concentration range of the type-B gelatin and formic acid solution combination. All scale bars = 50 μm.

**Figure 4:**
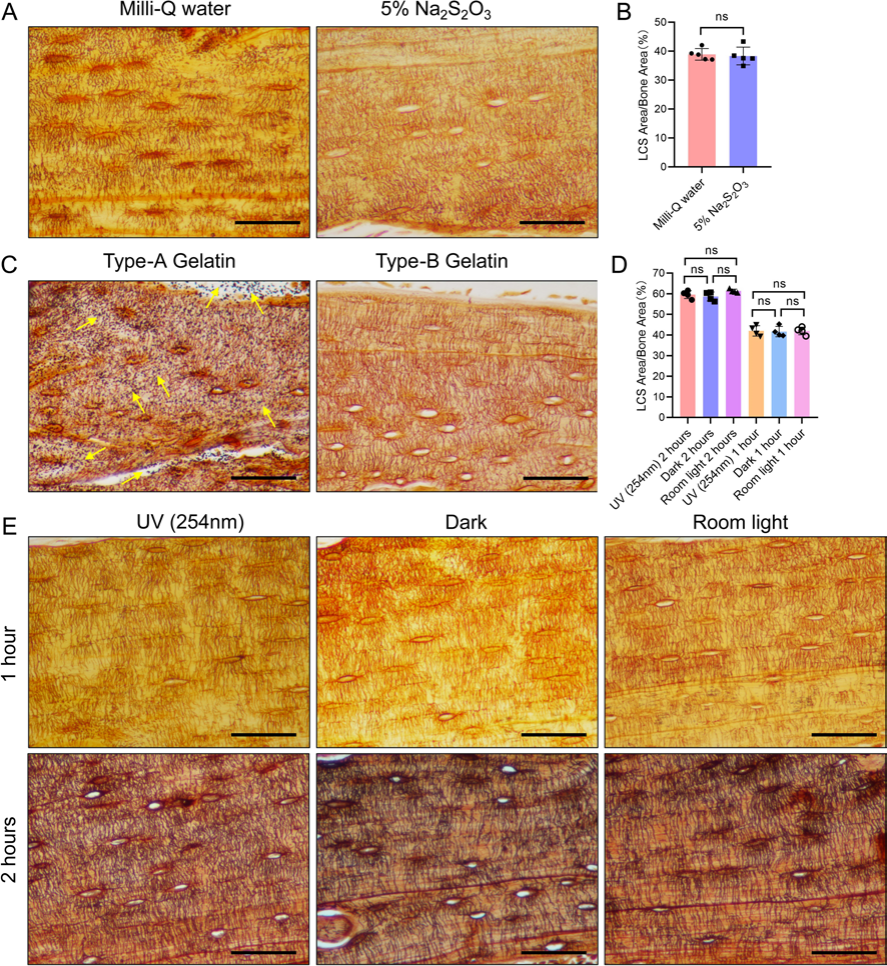
Effects of Three Different Conditions on Silver Nitrate Staining of LCS. **(A)** Effects of post-reaction Mill-Q water washing and 5% sodium thiosulfate washing on LCS staining. Scale bars = 50 μm. **(B)** Comparison of staining effects on osteocyte LCS between Milli-Q water and 5% sodium thiosulfate, analyzed using an unpaired two-tailed Student’s t-test. Data are presented as mean ± SD. ns p > 0.05, n = 5. **(C)** Effects of type-A and type-B gelatin on LCS staining. Yellow arrows indicate the presence of a large amount of black granular precipitates on the tissues after staining with type-A gelatin. Scale bars = 50 μm. **(D)** Comparison of staining effects on osteocyte LCS under three different lighting conditions, analyzed using one-way ANOVA. Data are presented as mean ± SD. ns p > 0.05, n = 4. **(E)** Effect of different light conditions (UV, room light, or dark) on LCS staining. Scale bars = 50 μm.

**Figure 5:**
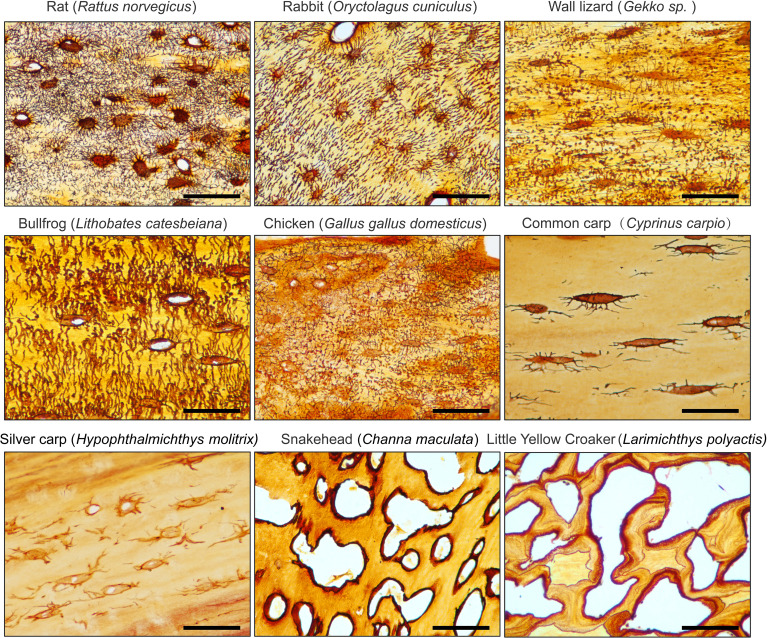
1 mol/L Silver Nitrate solution effectively stains the osteocyte LCS across different animal species. All Scale bars = 50 μm.

**Figure 6:**
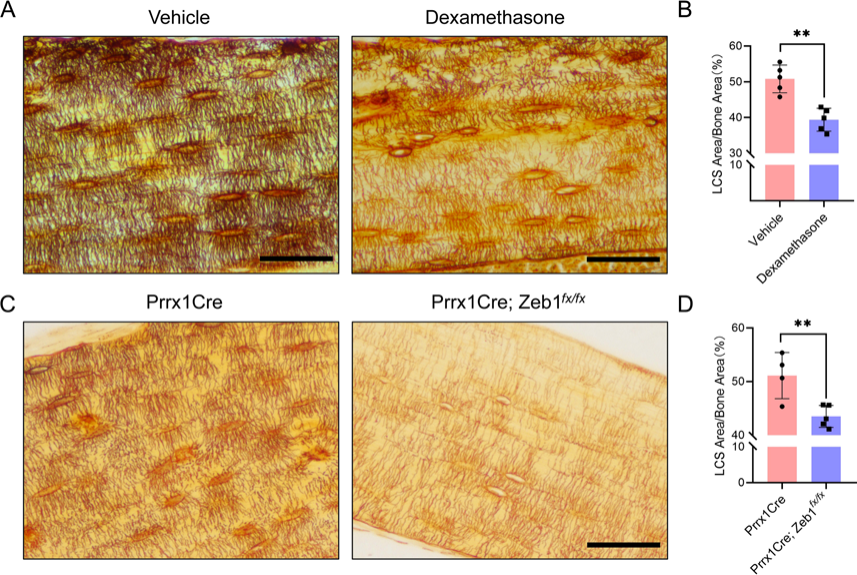
LCS Staining of Cortical Bone in Two Mouse Models Using 1 mol/L Silver Nitrate. **(A)** Cortical bone LCS was significantly reduced in glucocorticoid-induced bone growth retardation mice compared to vehicle-treated mice. Scale bars = 50 μm. **(B)** Comparison of osteocyte LCS density between glucocorticoid-induced bone growth retardation mice and vehicle mice, analyzed using an unpaired two-tailed Student’s t-test. Data presented as mean ± SD. **p < 0.01, n = 5. **(C)** Cortical bone LCS was significantly decreased in Prrx1Cre;Zeb1*^fx/fx^* mice compared to Prrx1Cre mice. Scale bars = 50 μm. **(D)** Comparison of osteocyte LCS density between Prrx1Cre;Zeb1^fx/fx^ mice and Prrx1Cre mice, analyzed using an unpaired two-tailed Student’s t-test. Data are presented as mean ± SD. **p < 0.01, n = 4 or 5.

**Figure 7:**
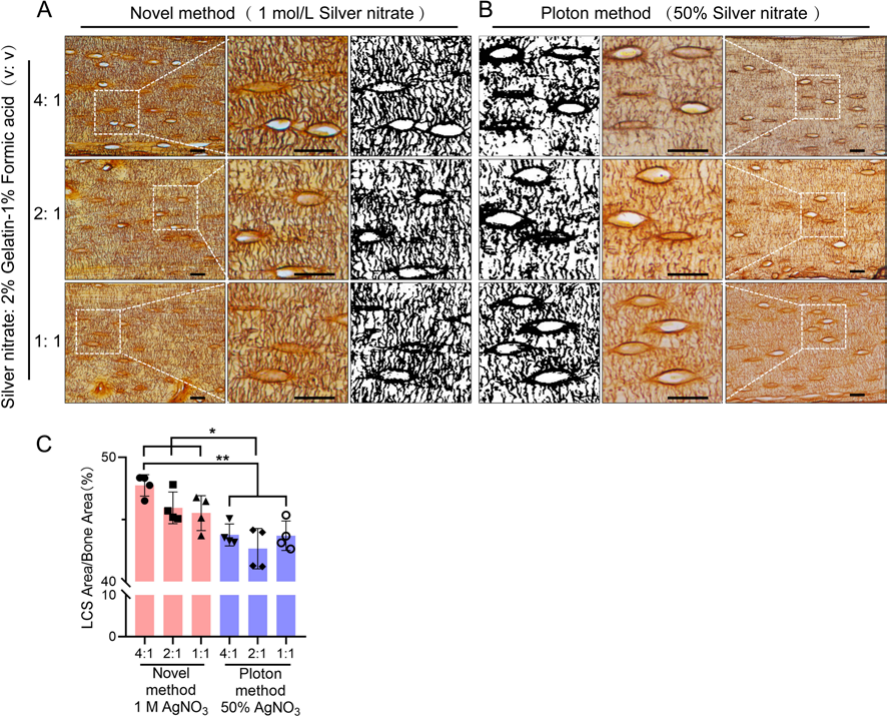
Comparison of the Staining effects of 1 mol/L Silver Nitrate and 50% Silver Nitrate on LCS. **(A)** Staining using 1 mol/L Silver Nitrate and 2% gelatin-1% formic acid at different volume ratios (4:1, 2:1, 1:1), with the third column showing binarized images. Scale bars = 20 μm. **(B)** Staining using 50% Silver Nitrate and 2% gelatin-1% formic acid at different volume ratios (4:1, 2:1, 1:1), with the first column showing binarized images. Scale bars = 20 μm. **(C)** Comparison of staining effects on osteocyte LCS density between the Novel method and the Ploton method, analyzed using one-way ANOVA. Data are presented as mean ± SD. *p < 0.05; **p < 0.01, n = 4.

In **Figures 2**, **3**, **4C**, **5**, each staining condition was repeated on at least two paraffin bone sections, with 3 to 5 fields of the fully developed cortical bone region taken from each section. In **Figure 4A**, each staining condition was repeated on five paraffin bone sections (n = 5), with at least 3 to 4 fields of the fully developed cortical bone region taken from each section. In **Figure 4E**, each staining condition was repeated on four paraffin bone sections (n = 4), with at least 3 to 4 fields of the fully developed cortical bone region taken from each section. In **Figures 6A, C**, femurs from 4 to 5 mice (n = 4 or 5) were isolated, sectioned, and stained, with at least 3 to 4 fields of the fully developed cortical bone region taken from each stained bone section. In **Figures 7A, B**, each staining condition was repeated on four paraffin bone sections (n = 4), with at least 3 to 4 fields of the fully developed cortical bone region taken from each section. Images were quantitatively analyzed using ImageJ software (NIH, USA) to obtain the ratio of LCS area to bone area (%), as described above. Data normality was assessed using the Shapiro-Wilk test in GraphPad Prism 8.0.1 software. For normally distributed data, statistical comparisons were performed using either one-way ANOVA or an unpaired two-tailed Student’s t-test. The data are presented as means ± standard deviation, represented by error bars. A p-value of < 0.05 was considered statistically significant.

## 3 Results

### 3.1 A 1 mol/L or 0.5 mol/L silver nitrate solution is an effective stain for osteocyte LCS

In preliminary experiments, we unexpectedly found that a 1 mol/L silver nitrate solution could effectively stain the osteocyte lacunae-canalicular system (LCS). Building on this discovery, we systematically evaluated the effects of varying silver nitrate concentrations (3 mol/L, 2 mol/L, 1 mol/L, 0.5 mol/L, 0.25 mol/L, and 0.1 mol/L) combined with a 2% type B gelatin and 1% formic acid solution at different volume ratios (4:1, 2:1, 1:1, 1:2, and 1:4). Staining was performed under UV light for either 1 or 2 hours, followed by termination with Milli-Q water (**Figure 2**).

Staining results indicated that under 254 nm UV light for 1 hour (**Figure 2A**), the 1 mol/L silver nitrate solution with 2% gelatin and 1% formic acid at ratios of 4:1, 2:1, and 1:1, as well as the 0.5 mol/L silver nitrate solution at a 4:1 ratio, produced clear and effective LCS staining compared to other combinations. When stained for 2 hours under 254 nm UV light (**Figure 2B**), the effective staining range expanded to include: 3 mol/L silver nitrate with 2% gelatin and 1% formic acid at ratios of 1:1, 1:2, or 1:4; 2 mol/L silver nitrate with 2% gelatin and 1% formic acid at ratios of 2:1, 1:1, 1:2, or 1:4; 1 mol/L silver nitrate at ratios of 4:1, 2:1, or 1:1; and 0.5 mol/L silver nitrate at ratios of 4:1 and 2:1.

Regardless of the staining duration (1 or 2 hours), the use of 3 mol/L silver nitrate with 2% gelatin and 1% formic acid at ratios of 4:1 and 2:1, or 2 mol/L silver nitrate at a 4:1 ratio, resulted in unclear staining and a darker background. This led to intermittent staining patterns, with fewer LCS stained compared to those stained with the 1 mol/L silver nitrate solution. Additionally, silver nitrate concentrations of 0.25 mol/L and 0.1 mol/L were ineffective in staining the osteocyte LCS under the same conditions. These findings suggest an optimal range of silver nitrate concentration for effective LCS staining; both excessively high and low concentrations failed to produce clear staining and accurate quantification of the LCS.

Based on the qualitative assessment of LCS staining, our data indicate that a 1 mol/L silver nitrate solution combined with 2% gelatin and 1% formic acid at ratios of 4:1, 2:1, or 1:1, or a 0.5 mol/L silver nitrate solution at a 4:1 ratio, under 254 nm UV light for 1 hour, can clearly and effectively stain the osteocyte LCS (as indicated by the dashed blue box in **Figure 2**).

### 3.2 A 1 mol/L silver nitrate solution, when combined with gelatin concentrations ranging from 0.05% to 0.5% (w/v) and formic acid concentrations from 0.05% to 5% (v/v), or with 1%-2% (w/v) gelatin and 0.1% to 2% (v/v) formic acid, effectively stains osteocyte LCS

After establishing the effective range of silver nitrate, we evaluated the impact of varying concentrations of type-B gelatin (0%-5%, w/v) and formic acid (0%-10%, v/v) on LCS staining, using a 1 mol/L silver nitrate solution and a gelatin-formic acid solution at a 2:1 volume ratio. The staining was conducted under 254 nm UV light for 1 hour, followed by termination with Milli-Q water (**Figure 3**).

The results indicated that silver nitrate alone could not stain LCS when both gelatin and formic acid concentrations were zero. When the gelatin concentration was zero and formic acid was ≥ 0.05%, LCS structures were visible under the microscope after staining, but a significant number of black granular precipitates adhered to the tissue, rendering the data unusable. In the absence of formic acid, silver nitrate failed to stain LCS at gelatin concentrations of 0.05% or higher. Furthermore, at a specific formic acid concentration (≥ 0.05%), the intensity of LCS staining decreased as gelatin concentration increased. Conversely, at a specific gelatin concentration (≥ 0.05%), the intensity of LCS staining initially increased and then decreased with increasing formic acid concentration.

In summary, the findings demonstrated that solution ratios with gelatin concentrations ranging from 0.05% to 0.5% and formic acid concentrations from 0.05% to 5%, as well as 1%-2% gelatin and 0.1% to 2% formic acid, resulted in the clear LCS staining, as indicated by the dashed blue box in **Figure 3**.

### 3.3 Optimal staining of the LCS can be achieved using type-B gelatin, with staining conducted under UV light for 1 hour or in room light (or in the dark) for 90 minutes, followed by termination of the reaction with Milli-Q water

Notable differences exist in the silver nitrate staining methods reported across various studies, particularly regarding the type of gelatin used (type A (19) or type B [20]), lighting conditions (UV, regular room light, or dark [17]), and reaction termination methods (Milli-Q water (15) or 5% sodium thiosulfate [16]). To evaluate the effects of these factors on LCS staining, we employed a 1 mol/L silver nitrate solution with 2% gelatin and 1% formic acid in a 2:1 volume ratio, staining under UV light for 1 hour (**Figure 4**).

The results indicated that tissues terminated with Milli-Q water exhibited clearer contrast and brighter staining compared to those terminated with 5% sodium thiosulfate (**Figure 4A**). However, no significant difference in the density of LCS staining was observed between the two termination methods (**Figure 4B**). When different types of gelatin were tested, type-A gelatin resulted in heavy black particulate deposits (**Figure 4C**, indicated by yellow arrows), while type-B gelatin produced a clear background without such deposits (**Figure 4C**). This suggests that the black particulate deposits observed in some studies may be due to the improper selection of gelatin type (19, 21).

Staining under different light conditions for one hour revealed that all three conditions (UV, regular room light, and dark) resulted in clearer LCS staining (**Figure 4E**). While no significant difference in LCS staining density was observed between the UV condition and regular room light (or dark) after one hour (**Figure 4D**), the UV condition produced deeper staining of the LCS compared to regular room light or dark under the same duration. After two hours of staining, the staining depth under regular room light and dark was comparable to that achieved with UV light (**Figure 4E**). Despite no noticeable difference in LCS staining density across the three light conditions after two hours (**Figure 4D**), the LCS background was slightly darker, and the contrast was less clear compared to one hour of staining.

Based on these observations, we conclude that staining for 60 minutes under UV light or for 90 minutes under regular room light or in the dark yields the most distinct staining. Therefore, optimal LCS staining on paraffin sections can be achieved using type-B gelatin, with staining for 1 hour under UV light or 90 minutes under regular room light or in the dark, followed by termination of the reaction with Milli-Q water.

### 3.4 A 1 mol/L silver nitrate solution effectively stains the LCS across different taxa

To assess the generalizability of the optimized staining method, we performed silver nitrate staining of LCS on skeletal samples from various species. The staining solution was prepared by mixing 1 mol/L silver nitrate with 2% type-B gelatin and 1% formic acid in a 2:1 volume ratio. The samples were reacted under UV light for 1 hour, followed by termination with a Milli-Q water wash (**Figure 5**).

The results showed that the optimized method effectively stained LCS in mammals (e.g., rat, rabbit), amphibians (e.g., bullfrog), birds (e.g., chicken), reptiles (e.g., wall lizard), and fishes (e.g., common carp, silver carp, snakehead, and little yellow croaker). However, the number of osteocytes in the caudal vertebrae of teleosts, such as common carp (*Cyprinus carpio*) and silver carp (*Hypophthalmichthys molitrix*), was notably low, and the LCS was sparsely present. In contrast, in the caudal vertebrae of other teleosts, such as snakehead (*Channa maculata*) and little yellow croaker (*Larimichthys polyactis*), silver nitrate staining revealed that mature vertebrae in these species were completely devoid of osteocytes and canalicular structures, with only lacunae-like structures visible. This suggests that teleosts generally lack mature osteocyte dendritic structures and a well-developed LCS, likely as an adaptation to their buoyant aquatic environments, as shown by phylogenetic studies of acellular bone (22, 23).

Thus, our novel silver nitrate method demonstrated broad applicability to skeletal tissues from various animal groups, highlighting its potential for comparative evolutionary biology studies on osteocytes and LCS systems across species.

### 3.5 A 1 mol/L silver nitrate solution effectively stains osteocyte LCS in both pathological and transgenic mouse models

To assess the potential utility of the optimized method in pathological models, we performed silver nitrate staining on bone samples from two mouse models: a glucocorticoid-induced bone growth retardation model and a transgenic mouse model (Prrx1Cre; Zeb1^fx/fx^) that overexpresses the Zeb1 transcription factor specifically in bone mesenchymal stem cells, exhibiting a phenotype characterized by pathological cartilage remnants in the cortical bone region of adult mice. The staining solution was prepared by mixing 1 mol/L silver nitrate with 2% type-B gelatin and 1% formic acid in a 2:1 volume ratio, and the reaction was under UV light for 1 hour and terminated by Milli-Q water washing (**Figure 6**).

The staining results showed that the optimized method effectively stained LCS in both models (**Figure 6**). The density of LCS was significantly reduced in the glucocorticoid-treated mice (**Figures 6A, B**) and Prrx1Cre; Zeb1^fx/fx^ mice (**Figures 6C, D**) compared to control animals. Notably, the staining results from Prrx1Cre; Zeb1^fx/fx^ mice indicated that Zeb1 overexpression in bone mesenchymal stem cells might impair the differentiation of mesenchymal stem cells into osteoblasts and osteocytes, leading to developmental disorders of the cortical bone and osteocyte LCS. These findings further suggest that our optimized staining method can effectively identify changes in LCS in common or new animal pathology models.

### 3.6 A 1 mol/L silver nitrate solution stains osteocyte LCS more effectively than the Ploton method

Next, we compared the optimized method using a 1 mol/L silver nitrate with the classical Ploton silver nitrate staining method, which uses 50% silver nitrate (2.943 mol/L), for staining LCS. We tested different concentrations of silver nitrate solutions (1 mol/L and 50%), combined with a 2% Type-B gelatin and 1% formic acid solution at varying ratios (4:1, 2:1, and 1:1). The reactions were carried out under UV light for 1 hour, followed by washing with Milli-Q water (**Figure 7**).

The results showed that the density of LCS stained with 1 mol/L silver nitrate was significantly higher than that stained with 50% silver nitrate, particularly when the volume ratio of silver nitrate solution to 2% gelatin-1% formic acid solution was 4:1 or 2:1. The LCS stained with 1 mol/L silver nitrate appeared more intact and clear (**Figure 7A**), whereas the LCS stained with 50% silver nitrate was more intermittent, irregular, and exhibited blurred contrast (**Figure 7B**).

In addition, statistical analysis shows that when using the classic Ploton staining solution ratio (i.e., a 2:1 volume ratio of 50% silver nitrate to 2% gelatin-1% formic acid solution), the LCS density stained is significantly lower than that stained with a 1 mol/L silver nitrate and 2% gelatin-1% formic acid solution at volume ratios of 4:1, 2:1, and 1:1 (**Figure 7C**). This result demonstrates the superior staining effectiveness of 1 mol/L silver nitrate compared to the Ploton method, which uses 50% silver nitrate.

## 4 Discussion

In this study, we developed and optimized a novel silver nitrate staining method for characterizing and quantifying the osteocyte LCS. Our results demonstrate that this optimized method, utilizing 1 mol/L silver nitrate, stains and quantifies the osteocyte LCS more efficiently than the classical Ploton method, which uses 50% silver nitrate.

Impregnation staining with a high concentration of silver nitrate (50%, w/v) is the most widely used technique for characterizing and quantifying the osteocyte LCS. This method was first reported by Goodpasture and Bloom in 1975 for staining chromosomal nucleolus organizer regions (NORs) (24), adapted from Howell et al.’s study published the same year, which focused on staining the satellite regions of human chromosomes with a 10% silver nitrate solution (25). In 1980, Howell and Black simplified Goodpasture and Bloom’s three-step staining method into a single step, improving the reproducibility and operability of the technique (15). Notably, the solutions used in their one-step method—50% silver nitrate in deionized water (Solution 1) and 2% gelatin in 1% formic acid (Solution 2), in a 2:1 volume ratio—remain unchanged today, and the incorporation of gelatin, a protective colloid, helped control the silver staining process and reduce background staining caused by the intense reduction of silver nitrate. In their 1986 study, Ploton et al. lowered the reaction temperature from 70°C to 20°C and used a 5% sodium thiosulfate solution to terminate the staining process (16), whereas Howell and Black’s method only used deionized water for washing out the staining mixture. In these studies, the staining duration was brief, ranging from a few seconds to a few minutes, as the staining was conducted on chromosomes in metaphase during mitosis.

According to the available literature, Chappard et al. first applied Ploton method in 1996 to stain the nucleolus organizer regions (NORs) of cells in undecalcified, MMA-embedded bones (17, 26). During this process, they accidentally discovered that the method also provided excellent staining of the osteocyte LCS. It was Chappard et al. who first determined that the optimal staining time for the osteocyte LCS was “55 minutes at room temperature in the dark,” followed by washing the slides with a 5% sodium thiosulfate solution. These conditions—both the staining reaction and the termination procedure—have since become the standard protocol for staining the osteocyte LCS. In subsequent studies, Chappard et al. adapted this method in staining the osteocyte LCS in paraffin-embedded bone samples after decalcification (27, 28).

Over the last two decades, there has been a growing recognition of the essential role osteocytes play in bone remodeling, the remodeling of the lacunar-canalicular system, and the development of osteocyte dendrites (29–32). Researchers have also adopted the staining solutions first described by Howell and Black, along with the 5% sodium thiosulfate solution for termination and room temperature reaction conditions introduced by Ploton et al., as well as the reaction time and dark conditions outlined by Chappard et al. This method is widely recognized in osteocyte research as Ploton silver staining (18) or the Ploton impregnation method (28).

In our preliminary study, we encountered difficulties in obtaining sufficient silver nitrate powder to prepare a 50% silver nitrate solution for osteocyte biology studies due to safety regulations. Instead, we utilized a 1 mol/L silver nitrate solution, which is readily available for chemical titrations, as a substitute for the 50% solution in staining the osteocyte LCS. Surprisingly, this solution effectively and clearly stained the osteocyte LCS. This finding prompted us to optimize the classical Ploton method by using a lower concentration of silver nitrate solution, thereby enabling more efficient and environmentally friendly staining and quantification of the osteocyte LCS.

Based on this idea and using the classical Ploton method as a reference, we evaluated several factors that could influence the effectiveness of silver nitrate staining. Consequently, we selected six key conditions for study: silver nitrate concentration, the concentrations of the gelatin-formic acid combination, the volume ratio of silver nitrate to gelatin-formic acid solution, type of gelatin, light conditions, and termination conditions. Our results demonstrated that, for 4 μm paraffin bone sections, optimal staining was achieved with a 1 mol/L silver nitrate solution combined with a gelatin-formic acid solution at various concentrations (0.05%-0.5% type-B gelatin and 0.05%-5% formic acid, or 1%-2% type-B gelatin and 0.1%-2% formic acid) in volume ratios of 4:1, 2:1, or 1:1, or a 0.5 mol/L silver nitrate solution at a 4:1 ratio. The staining process was carried out for 1 hour under UV light in a biosafety cabinet or 90 minutes under regular room light (or dark), followed by washing with Milli-Q water to terminate the reaction.

Next, we applied the optimized, low-concentration, cost-effective, and environmentally friendly staining method to the osteocyte LCS in bone samples from various animal species. Our experiments demonstrated that this method effectively provides clear contrast staining of the osteocyte LCS in the bones of mammals, amphibians, fish, birds, and reptiles. Notably, we observed that teleost vertebrae, compared to those of other species, contained significantly fewer mature osteocytes and exhibited a notably lower number and density of LCS. This finding suggests that the optimized staining method could serve as a powerful tool for comparative evolutionary studies of osteocyte and LCS morphogenesis across different vertebrate taxa.

In addition, the optimized method effectively stains the LCS in both glucocorticoid-induced bone growth retardation mice and Zeb1 overexpression transgenic mice (Prrx1Cre;Zeb1*^fx/fx^*). In the model group, the density of the LCS in the femoral cortical bone was significantly lower than in the control group. Notably, in the Prrx1Cre;Zeb1*^fx/fx^* mice, we observed not only a marked reduction in LCS density but also extensive cartilage remnants in the cortical bone (Alcian Blue-Safranin O staining, data not shown). These findings, for the first time, suggest that the Prrx1Cre;Zeb1*^fx/fx^* transgenic mice may serve as a new model for cortical bone development, providing a valuable platform for investigating the expression patterns and underlying mechanisms of transcription factors involved in cortical bone development and osteocyte maturation.

Finally, we compared the optimized method (1 mol/L silver nitrate) with the classic Ploton method (50% silver nitrate) for LCS staining. Statistical analysis revealed that the 1 mol/L silver nitrate staining resulted in significantly higher LCS density compared to the 50% silver nitrate method. This finding highlights a potential limitation of the Ploton method: the 50% silver nitrate solution may not fully reveal osteocyte LCS in bones. The strong reduction reaction of the high-concentration silver nitrate could damage the LCS or fail to adequately stain osteocyte LCS in bones, potentially leading to an underestimation of osteocyte LCS density or number. Although no direct comparison was made in this study, we also observed that the optimized method yielded better results than another technique using the Silver-Protein method (or Bodian’s Protargol-S method) for staining osteocyte LCS (33, 34). We recommend that researchers studying osteocytes carefully consider the inherent limitations of the Ploton method and Silver-Protein method, and critically evaluate the novel optimized method presented in this paper.

Currently, several histological staining methods are available for visualizing osteocyte LCS, including silver nitrate staining (20), silver-protein staining (33, 34), basic fuchsin staining (35), rhodamine 6G staining (36–38), and fluorescein isothiocyanate (FITC) staining (39, 40). The procedure for silver protein staining is complex and time-consuming, and its staining effect and resolution are not as clear as those achieved with silver nitrate, making it less commonly used in the research field of osteocyte LCS. Basic fuchsin staining, rhodamine 6G staining, and FITC staining typically require special methyl-methacrylate (MMA) embedding of the bone, along with the use of expensive, specialized hard tissue microtomes and grinding equipment for sectioning. Although these methods can yield clear two-dimensional or three-dimensional imaging of LCS through confocal laser scanning microscopy (CLSM), their time-consuming and complex sample processing makes them less straightforward than paraffin sectioning, and they do not provide better resolution than silver nitrate staining. Emerging techniques, such as resin cast etching (RCE), scanning electron microscopy (SEM), serial focused ion beam/scanning electron microscopy (FIB/SEM), transmission electron microscopy (TEM), and synchrotron-radiation computed tomography (SR-μCT), greatly enhance LCS imaging and generate new insights (3, 41, 42). However, these methods rely on expensive instruments and are not suitable for routine laboratory histological studies. Therefore, the novel silver nitrate staining method for osteocyte LCS reported in this article, achieved through systematic optimization, holds significant importance and value compared to these techniques.

Undoubtedly, our method has certain limitations and potential for further optimization. We have found that increasing the incubation temperature (for example, from room temperature to 37°C) can accelerate the staining process; therefore, comparing the effects of different temperatures on staining outcomes could be significant for further optimization. Additionally, due to the high density of osteocyte LCS that have evolved in mammalian skeletal systems, our optimized staining method still does not fully reveal the complex structure of the LCS necessary for effective mathematical analysis and physical simulation of this intricate network. Therefore, in further optimizing this method, If the revolutionary expansion microscopy technique developed by Boyden et al. could be applied to this staining process—either by performing isotropic expansion on tissue sections after silver nitrate staining or by conducting whole-tissue expansion following decalcification of the bone before sectioning and silver nitrate staining—we believe that this approach would yield clearer LCS staining results than current methods and provide new insights into LCS structure in the future (43–45).

## 5 Conclusion

In conclusion, this study represents the first systematic optimization of a staining method that has been established for nearly half a century and utilized in osteocyte research for almost 30 years. The novel optimized method uses a 1 mol/L silver nitrate solution combined with a gelatin-formic acid solution at various concentrations (0.05%-0.5% type-B gelatin and 0.05%-5% formic acid, or 1%-2% type-B gelatin and 0.1%-2% formic acid) in volume ratios of 4:1, 2:1, or 1:1, or a 0.5 mol/L silver nitrate solution at a 4:1 ratio. The staining process was carried out for 1 hour under UV light or 90 minutes under regular room light (or dark), followed by washing with Milli-Q water to terminate the reaction. This novel method effectively stains LCS across different species and disease models, demonstrating greater number and density of stained LCS compared to the classical Ploton method. We believe that our optimized method will advance osteocyte biology research and provide a valuable tool for studying the adaptive evolution of osteocyte LCS morphology and function.

## Data Availability

The raw data supporting the conclusions of this article will be made available by the authors, without undue reservation.

## Author’s note

Wu Jinlian and Wang Libo dedicate their first scientific discovery to their beloved parents and daughters, Luna and Lina, in gratitude for their cherished love and unwavering support throughout their academic pursuit.
